# Health-Seeking Behaviour and Its Determinants Following the COVID-19 Pandemic

**DOI:** 10.7759/cureus.52225

**Published:** 2024-01-13

**Authors:** Arham Yahya Rizwan Khan, Mashal Aziz Rana, Dur e Shewar Naqvi, Amina Asif, Fizza Najeeb, Sajida Naseem

**Affiliations:** 1 Shifa College of Medicine, Shifa Tameer-e-Millat University, Islamabad, PAK; 2 Family and Community Medicine, Shifa Tameer-e-Millat University, Islamabad, PAK

**Keywords:** health education & awareness, covid 19 impact of lockdown, global pandemic, covid-19, health seeking behavior

## Abstract

Objective: The purpose of this study was to determine the change in behaviour of individuals towards any health issues they faced after the coronavirus disease 2019 (COVID-19) pandemic and to compare the health-seeking behaviour of people who were infected by the virus and those who were not infected.

Methods: A cross-sectional study was conducted among 400 participants visiting Shifa International Hospital, Islamabad, Pakistan, and Pakistan Institute of Medical Sciences Hospital, Islamabad, Pakistan. Data was collected through a pilot-tested questionnaire and analyzed using IBM SPSS Statistics for Windows, Version 26.0 (Released 2019; IBM Corp., Armonk, New York, United States).

Results: In 286 participants (71.6%), health-seeking behaviours were significantly altered by the COVID-19 pandemic. Overall, this research showed that COVID-19 was linked to poor health-seeking behaviour.

Conclusion: Most of the participants’ health-seeking behaviours were significantly altered by the COVID-19 pandemic. A significant change in how people behaved towards any health problem was reported. As a result, public awareness campaigns should focus on delivering more information about COVID-19 to promote their health-seeking behaviour.

## Introduction

Health-seeking behaviour is defined as a measure of action taken by a diseased individual to gain access to an effective therapeutic approach [1]. The promotion of efficient care-seeking behaviour has long been the prime focus of health promotion missions across the globe. Such programs are renowned for enhancing the degree of public awareness and education regarding a plethora of preventable morbidities [2]. The characteristics of an ideal health-seeking behaviour include self-supervision of an individual’s bodily symptoms, modulating the plan of action in line with their degree of severity, and seeking timely professional healthcare. The overall extent of health-seeking behaviour can be altered by a multitude of factors such as socioeconomic profile, general physical health, disease severity, and geopolitical, environmental, and cultural factors [3]. However, since 2020, the health-seeking behaviour of the public has been greatly impacted by the emergence of one of the greatest pandemics of modern times, i.e., coronavirus disease 2019 (COVID-19).

The severe acute respiratory syndrome coronavirus 2 (SARS‑CoV‑2) outbreak was declared a pandemic by the World Health Organization (WHO) in March 2020, and the total number of mortalities as of December 31, 2023, was 7,010,568 [4]. This viral infection left a mark on our physical health as well as the socioeconomic and cultural aspects of our lives. Due to nationwide lockdowns and with most of the hospitals flooded by COVID-19 patients, health-seeking behaviour was adversely impacted by the pandemic [5]. One survey conducted by Wong et al. showed that the cumulative frequency of outdoor check-ups dropped sharply during the COVID-19 pandemic in Hong Kong [6]. This can be attributed to rising fear and distress among the masses about contracting an acute respiratory infection [7]. In line with this, it has been feared that thousands of patients suffering from chronic disease might be lost to follow-up [8]. Arshad et al. revealed that COVID-19 isolation led to a gradual increase in the frequency of self-medication among individuals, another hallmark of low-quality health-seeking behaviour [9]. In contrast, mass awareness has also allowed global health institutions to witness an increasing trend in the adoption of preventive measures against COVID-19 and other related respiratory illnesses. Moreover, healthcare consultation has been significantly promoted in some regions, which is likely due to a higher degree of health consciousness and an increased level of public education [10].

It is imperative that the medical community has current knowledge regarding the magnitude of the impact of the COVID-19 pandemic on general health-seeking behaviour because it is pivotal to the inception of a well-informed healthcare system. Therefore, the purpose of this study is to assess the changes in health-seeking behaviour in the post-pandemic era.

## Materials and methods

This cross-sectional study was conducted at two tertiary care hospitals in Islamabad, Pakistan. These hospitals were Shifa International Hospital and Pakistan Institute of Medical Sciences (PIMS). Approval was obtained from the Institutional Review Board and Ethics Committee of Shifa International Hospital (approval number: 028-22) and the Hospital Ethics Committee of PIMS (approval number: ECPIMS/23/02). This study was conducted according to the ethical principles of the Declaration of Helsinki.

The population under study were the patients presenting to the outpatient departments of both hospitals mentioned above. To calculate the sample size, the OpenEpi online sample size calculator was used (www.OpenEpi.com), and the minimum sample size was determined to be 400 with a confidence level of 95% and a margin of error of 5%. Participants were selected using a non-probability purposive sampling technique. Patients aged 18 years or older were included in the study and patients who did not give consent to take part in the study or did not understand the local language, Urdu, were excluded from the study.

Data was collected over a period of six months from December 2021 to May 2022. Written informed consent was obtained from all participants after which they were asked to anonymously fill out a questionnaire in the Urdu language. The self-designed questionnaire comprised 19 multiple-choice questions with each question having a minimum of two answer choices to choose from and a maximum of six options. It included questions about age, gender, level of education, socioeconomic background, history of chronic disease, history of COVID-19 illness, and questions related to health-seeking behaviour before and during the COVID-19 pandemic. The questionnaire was designed to take a maximum of 15 minutes to answer by each participant. Prior to the start of the study, the questionnaire was pre-tested on a population of 20 patients from each hospital to identify any ambiguity in the questions. Cronbach's alpha was used to measure internal consistency. One question was removed during this process and the final Cronbach's alpha value was 0.82 suggesting relatively high internal consistency as 0.70 or higher was considered acceptable. A total of 400 people from both hospitals took part in this study.

The questionnaire was translated into English by an expert translator for data analysis. Data was analysed using IBM SPSS Statistics for Windows, Version 26.0 (Released 2019; IBM Corp., Armonk, New York, United States). Continuous data were presented as means with standard deviation and categorical data were presented as frequencies and percentages. Chi-square tests were used to identify associations between categorical variables. A p-value <0.05 was considered as significant. Bivariate and multivariate logistic regression analyses were also performed to determine significant predictors of outcomes with an estimation of the OR and 95% CI.

## Results

A total of 400 respondents were approached and involved in the study. Of these, 233 (58.3%) and 164 (41.8%) were from PIMS Hospital and Shifa International Hospital, respectively. Of the participants, half were male (n=201, 50.3%), and the majority were permanent residents of Pakistan (n=348, 87.0%). The mean age of the respondents was 29.11± 7.39 years. In this study, approximately 36.3% of the participants had graduate-level educational status. Moreover, those participants who had healthcare-related occupations accounted for 53.3% (n=213) of the participants. There were 128 (32.0%) participants who had a monthly income of less than 30,000 Pakistani Rupees (Table 1).

**Table 1:** Social and demographic characteristics of study participants PKR: Pakistani Rupee

Variables	Frequency	Percentage
Gender		
Male	201	50.3
Female	199	49.8
Permanent resident of Pakistan		
Yes	348	87
No	53	13
Educational status		
Primary	35	8.8
Matriculation	56	14
Intermediate	99	24.8
Graduate	145	36.3
Post-graduation	60	15
Uneducated	5	1.3
Occupation		
Healthcare-related	213	53.3
Non-health care related	187	46.8
Monthly income (PKR)		
<30,000	128	32
30,000-60,000	115	28.8
61,000-100,000	68	17
101,000-150,000	53	13.3
>150,000	34	8.9

Approximately 36% of the study participants (n = 144) had chronic illness. Moreover, approximately 42% (n =167) of the participants tested positive for COVID-19 previously. Just over half of the participants (n=206, 51.5%) had lost a friend or relative due to COVID-19. Additionally, more than half of the participants (n=220, 55.0%) reported that their primary mode of action when they experienced a health-related problem was to consult a well-trained physician (Table 2).

**Table 2:** Clinical characteristics of study participants COVID-19: coronavirus disease-19

Variables	Frequency	Percentage
Chronic illness		
Yes	144	36
No	256	64
Lost friend or relative due to COVID-19		
Yes	206	51.5
No	192	48.5
Primary mode of action for any health-related problem		
I look up my clinical symptoms online	94	23.5
Consult a well-trained physician	220	55
Consult an expert of alternative medicine	38	9.5
Discuss my symptoms with a pharmacist	30	7.5
Self-administer over-the-counter medications	18	4.5

Most of the participants’ (n=286, 71.6%) health-seeking behaviour was strongly altered by the COVID-19 pandemic. Meanwhile, 132 (33.0%) participants were less likely to visit a doctor/hospital during the COVID-19 pandemic for minor symptoms such as headache or diarrhoea. Only 73 (18.3%) participants did not miss their doctor’s appointments; one-third (n=133, 33.3%) of the participants missed two scheduled doctor’s appointments due to COVID-19 restrictions. The preferred method of taking medication before and after the COVID-19 pandemic was after consulting a physician, with 269 (67.3%) participants doing this before and 247 (61.8%) after the COVID-19 pandemic. Additionally, 148 (37.0%) participants self-medicated during the COVID-19 pandemic. The chi-square test revealed that there was a significant change in the behaviour of individuals towards any health issue they faced after the COVID-19 pandemic (p<0.0001) (Table 3).

**Table 3:** Change in behaviour of individuals towards any health issue they face after the COVID-19 pandemic COVID-19: coronavirus disease 2019

Variables	Frequency	Percentage
How has the COVID-19 pandemic altered behaviour?		
Very strongly	143	35.8
Strongly	143	35.8
Moderately	52	13
Mildly	50	12.5
Not affected at all	12	3
Likelihood of visiting a doctor/hospital during the COVID-19 pandemic for minor symptoms such as headache or diarrhoea		
Extremely likely	103	25.8
Very likely	122	30.5
Less likely	132	33
Very unlikely	43	10.7
Missed scheduled doctor’s appointments due to COVID-19 restrictions		
One	93	23.3
Two	133	33.3
>two	101	25.1
None	73	18.3
How did you take your medication to treat an illness before the COVID-19 pandemic?		
After consulting a physician	269	67.3
After consulting a pharmacist	62	15.5
After consulting family members or friends	47	11.8
After reading about it online	10	2.5
Based on some past experience	12	3
How did you take your medication to treat an illness after the COVID-19 pandemic?		
After consulting a physician	247	61.8
After consulting a pharmacist	60	15
After consulting family members or friends	58	14.5
After reading about it online	18	4.5
Based on some past experience	17	4.2
How often have you self-medicated yourself during the COVID-19 pandemic?		
Once	148	37
Twice	52	13
Three or more times	74	18.5
Never	126	31.5

**Figure 1:**
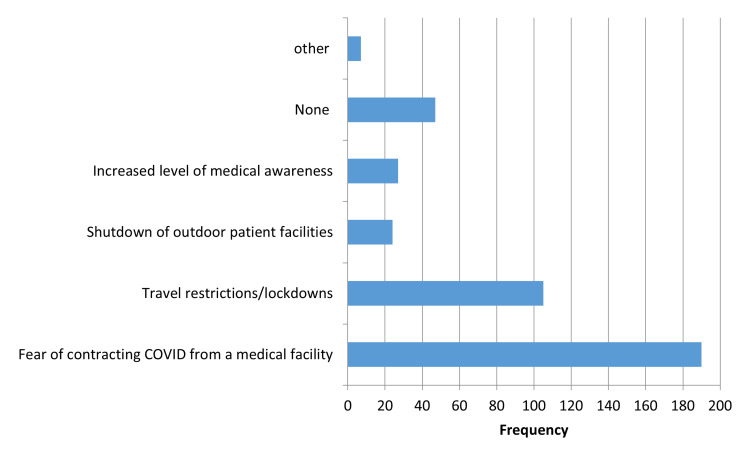
Contributing factors behind altered health-seeking behaviour during the pandemic COVID: coronavirus disease 2019

The COVID-19 pandemic affected health-seeking behaviour regarding medication in 278 (69.5%) participants. The most common contributing factors behind altered health-seeking behaviour during the pandemic were fear of contracting COVID-19 from a medical facility (n = 190; 47.5%), travel restrictions/lockdowns (n = 105; 26.3%), and increased level of medical awareness (n = 27; 6.8%) (Figure 1).

In the bivariate logistic regression analysis, educational status, occupation, and history of previous COVID-19 positive tests were significantly associated with changes in health-seeking behaviour due to the pandemic (p-value < 0.2). These variables were entered into the multivariable logistic regression model. Consequently, the multivariable binary logistic regression analysis revealed that only occupation was found to be significantly associated with health-seeking behaviour due to the pandemic.

The odds of participant behaviour being affected by COVID-19 were significantly higher (OR=1.71, 95%CI: 1.09, 2.69) among non-healthcare professionals than among healthcare professionals. Age, sex, social status, monthly income and other sociodemographic and clinical characteristics were not associated with changes in health-seeking behaviour due to COVID-19 (Table 4).

**Table 4:** Factors affecting health-seeking attitudes during the COVID-19 pandemic COVID-19: coronavirus disease 2019

Variables	Has COVID-19 affected health seeking behaviour?		OR (95% CI)	p-value	
	Yes	No	Crude Odds Ratio (COR)	Adjusted Odds Ratio (AOR)	
Educational status					
Primary	22	13	1	1	
Matriculation	41	15	0.62 (0.25-1.53)	0.59 (0.23-1.46)	0.251
Intermediate	71	28	0.67 (0.30-1.51)	0.66 (0.29-1.51)	0.327
Graduate	98	47	0.81 (0.38-1.75)	0.91 (0.42-2.0)	0.818
Post –graduation	45	15	0.56 (0.23-1.39)	0.67 (0.27-1.69)	0.396
Uneducated	1	4	6.77 (0.68-67.25)	6.04 (0.60-61.22)	0.128
Occupation					
Non-healthcare related professionals	161	52	1.85 (1.20-2.85)	1.71 (1.09-2.69)	0.020
Healthcare professionals	117	70	1	1	
Have you tested positive for COVID-19?					
Yes	125	42	1.56 (1.00-2.42)	1.54 (0.97-2.43)	0.067
No	153	80	1	1	

The health-seeking behaviour of those who tested positive for COVID-19 was impacted by the pandemic for 125 participants (75%). Moreover, the behaviour regarding medication in 119 participants who tested positive for COVID-19 was strongly affected by the pandemic. Bivariate logistic regression analysis revealed that the odds of participants’ behaviour being affected by COVID-19 among those who tested positive for COVID-19 was 1.56 times (95% CI 1-2.42) higher than that among their counterparts. However, there was no difference with multivariate logistic regression.

## Discussion

This study evaluated COVID-19's effects on patients' health-seeking behaviours and significantly adds to the limited collection of studies on patients' health-seeking behaviours during COVID-19, especially in Pakistan. The health-seeking behaviour of the majority of the participants (n=286, 71.6%) was significantly altered by the COVID-19 pandemic. However, owing to COVID-19 restrictions, 133 (33.3%) participants missed two planned doctor appointments. Additionally, after the COVID-19 pandemic, there was a significant change in how people behaved towards any health problem they encountered (p <0.0001). Overall, the current study showed that COVID-19 was linked to poor health-seeking behaviour.

Numerous earlier studies that revealed unhealthy health-seeking behaviour lend support to this. According to a study conducted in Ethiopia, COVID-19 had a significant effect on patients' health-seeking behaviour, which was poor in 39% of patients [11]. Similar research conducted in Singapore during the COVID-19 outbreak indicated that 40% of chronic patients missed follow-up appointments [12]. Fear of getting COVID-19 from a hospital was one of the most common reasons people changed their health-seeking behaviour during the pandemic.

This led to a tendency in the population to self-medicate, and according to the current study, 37% of the participants did so during the COVID-19 pandemic. According to this study, those participants who did not work in the healthcare industry had 1.7 times the likelihood of being affected by the pandemic compared to healthcare professionals. This may be the result of misinformation being spread to non-healthcare workers. During the rampant spread of COVID-19 around the world, many societies saw the emergence of false information, conspiracies, and widespread public doubts about what is happening [13]. Even though most of these rumours were swiftly disproven and shown to be false, the prevalence of false information and conspiracy theories on social media and in the news has changed society's attitudes towards seeking out healthcare. In addition, social media platforms and digital technologies made it possible for news media producers and users to share information quickly and easily across platforms. False and fake narratives frequently perform better than actual news in these online environments in terms of audience interaction and popularity [14]. As a result, rumours, conspiracy theories, and "alternative truths" frequently flourish in circumstances of high fear, low confidence, and low trust [15]. Furthermore, earlier research demonstrated a link between exposure to disease-related conspiracy theories and lower levels of trust in governmental and healthcare institutions [16]. This finding emphasizes the potentially damaging and far-reaching effects of misinformation and disinformation, including altered health-seeking behaviour. On the other hand, healthcare professionals had information and scientific knowledge regarding the properties of the virus and adequate knowledge about the virus compared to non-healthcare professionals, and they were less likely affected by the pandemic. Moreover, since they were front-line workers, they were constantly updated about the virus and the preventive measures; thus, most of the fear and contributing factors for the altered behaviour as well as conspiracy beliefs were handled better, leading to less influence of the pandemic on their health-seeking habits.

A total of 125 participants' health-seeking behaviours, among those who tested positive for COVID-19, were impacted by the pandemic in the current study. This is comparable to the study by Tian et al., which found that patients' health-seeking behaviours were greatly impacted by their prior interactions with COVID-19 patients [17]. These patients continue to experience significant physical and psychological problems even after being discharged.

After witnessing the deaths of other patients, seeing their clinical condition worsen, learning about the high death rate of COVID-19 patients worldwide through the media, and being separated from their loved ones, participants experienced fears of a difficult and painful death. Fear is an adaptive reaction to a possibly dangerous situation and is one of the key elements of psychological trauma. Fear of dying and a worsening of symptoms, according to a study, are some of the most significant emotional stresses experienced by COVID-19 patients [18]. Additionally, according to another study, COVID-19 patients constantly worry about their impending deaths, relapses, and unexpected complications [19]. These patients may dread reinfection because of their extreme psychological stress and generally negative experiences, which may affect how they seek medical attention. Furthermore, their fear of contracting COVID-19 while receiving treatment in COVID-19-designated receiving hospitals may alter their health-seeking behaviour. Thus, it is understandable that they put off getting care. However, this choice may be harmful and put many patients in danger of unfavourable results.

Limitations

This study has some limitations. This is a small study, thus affecting statistical power. Additionally, we note that this study covers only one year of the pandemic. Hence, it is possible that the results may be affected by the potential proximity to the most severe points of the pandemic. Despite these limitations, we believe this study provides a picture of the health-related behaviours of patients following the COVID-19 pandemic.

## Conclusions

Most of the participants’ health-seeking behaviours were markedly altered by the COVID-19 pandemic. A significant change in how people behaved toward any health problem was reported. Furthermore, the most common contributing factors behind altered health-seeking behaviour during the pandemic were fear of contracting COVID-19 from a medical facility, travel restrictions/lockdowns, and increased levels of medical awareness. As a result, public awareness campaigns should focus on delivering more information to promote health-seeking behaviour. Furthermore, it is critical to disseminate accurate information, particularly among non-healthcare-related professionals and previous COVID-19-positive patients, as their health-seeking behaviour has been highly affected by the pandemic.
